# Chemical Fractions and Availability of Zinc in Winter Wheat Soil in Response to Nitrogen and Zinc Combinations

**DOI:** 10.3389/fpls.2018.01489

**Published:** 2018-10-12

**Authors:** Hongen Liu, Peng Zhao, Shiyu Qin, Zhaojun Nie

**Affiliations:** Resources and Environment College, Henan Agricultural University, Zhengzhou, China

**Keywords:** nitrogen, soil available zinc, soil pH, soil zinc chemical fractions, winter wheat, zinc

## Abstract

Nitrogen (N) is critical for zinc (Zn) accumulation in winter wheat grain via enhancing Zn absorption into plant roots. This paper explored a possible mechanism for enhanced absorption of Zn in winter wheat by N combined with Zn application based on the Zn bio-availability in soil. A pot experiment with three N application rates (0.05, 0.2, and 0.4 g kg^-1^), two Zn application rates (0 and 10 mg kg^-1^), without and with plants was conducted. The results showed that high N (N_0.2_ and N_0.4_) combined with Zn (Zn_10_) application significantly increased the yield, yield components and Zn and N concentrations in winter wheat shoots and grain. The available Zn concentration in soil with and without plants was increased by N_0.2_Zn_10_ and N_0.4_Zn_10_ treatment at each growth stage. N_0.2_Zn_10_ and N_0.4_Zn_10_ treatment significantly decreased the pH in soil without plants but had different influences on the pH in soil with plants, which depended on the different N application rates and growth stages. Meanwhile, N_0.2_Zn_10_ and N_0.4_Zn_10_ treatment decreased the exchangeable Zn but increased loose organic-, carbonate- and Fe-Mn oxides-bound Zn concentrations in soil without plants. The exchangeable, loose organic- and carbonate-bound Zn concentrations in soil with plants was increased by N_0.2_Zn_10_ and N_0.4_Zn_10_ treatment at different growth stages. Different rates of N combined with Zn application influenced the proportion of Zn in different fractions in soil with and without plants at different growth stages. At Zn_10_, N_0.4_ treatment showed higher yield, N and Zn concentrations in shoot and grain, and available Zn concentration in soil, but lower pH in soil than N_0.2_ treatment. In addition, soil without plants had higher available Zn concentrations and lower pH than did the soil with plants. There were significant differences in Zn chemical fractions concentrations and proportions between the soils with and without plants at each growth stage. Therefore, combined influence of roots and the combination of N and Zn (especially N_0.4_Zn_10_ treatment) improved the bio-availability of Zn in soil via changing the soil pH and promoting the transformation and distribution of Zn in different fractions.

## Introduction

Zinc (Zn) is essential for human body and is involved in physiological and nutritional functions in human growth and development, including humoral and cellular immunity as well as the synthesis of proteins and nucleic acids (Bonaventuraa et al., 2015). Zn deficiency results in a series of problems for humans, especially infants, such as loss of appetite and digestion, growth retardation, and brain and immune system dysfunction (Prasad, 2014). Zn is also a necessary element in plants, and Zn deficiency in soil can decrease the yield and quality of crops, consequently threatening human health. Zn deficiency in the body can be resolved by human intervention, including dietary diversification, nutritional supplements, food intensification and biofortification (Graham et al., 2001). For areas with severe zinc deficiency, such as sub-Saharan Africa and South Asia, food crops in those developing countries are the main source of Zn in the human body. Thus, biofortification of grain crops with Zn by breeding and fertilization appears to be cost-effective and promising as an approach for improving Zn concentration in grain, thereby contributing to human health (Graham et al., 2001; Bouis et al., 2011; Velu et al., 2014).

As a source of both calories and protein, wheat is one of important crops in many countries (Cakmak, 2008). However, widespread low Zn concentrations in wheat lead to insufficient intake of Zn by populations for whom wheat is the staple food. The Zn concentrations in wheat grain in the Northern Winter Wheat Region of China range from 11.7 to 49.7 mg kg^-1^, with a mean value of 26.4 mg kg^-1^ (Zhang et al., 2007; Cao et al., 2010), which is well below the target value of 40–60 mg kg^-1^ (Ortiz-Monasterio et al., 2007; Cakmak, 2008). The reason for the low Zn concentration in crop grain is the low level of Zn supply in soil (Hotz and Brown, 2004). However, Zn deficiency in soil is common in China (Liu, 1994). Henan Province is the main area for wheat production in China, and more than 60% of soil has severe Zn deficiency, approximately 34% of the soil is possibly Zn deficient, and only 6% of the soil is Zn sufficient (Sun and Cai, 1984).

The Zn adsorption-desorption reactions between the solution and solid phases control Zn concentrations in soil solution and the availability of Zn to plants (Lindsay, 1991; Catlett et al., 2002), which depend on the pH, organic matter, soil minerals, and co-existing ions as well as the distribution into various fractions (Alloway, 2008). The Zn fractions in soil are often distinguished with regard to chemical binding characteristics, including exchangeable, organic matter-bound, carbonate-bound, Fe-Mn oxides and residual Zn (Tessier et al., 1979; Jiang et al., 1990). Exchangeable Zn is the most labile binding form and has the closest correlation with Zn uptake in plants (Chahal et al., 2005; Li et al., 2007). Organic matter-bound Zn is also available to plants due to the exchangeable sites for Zn in soil solid matrix provided and cation exchange capacity increased by organic matter (Khoshgoftarmanesh et al., 2018). When Zn is bound to carbonate or Fe-Me oxides in soil, this binding will reduce the bioavailability of Zn and will enhance the ability of rice to resist Zn stress (Shuman and Wang, 1997).

Plants have direct or indirect influences on the nutrients availability in diverse ways, such as the release of root exudates (Clemens et al., 2002; Udom et al., 2004). Root exudates in oat can dissolve the heavy metals bound to carbonate and oxides and can convert them to the exchangeable form, improving the availability of heavy metals (Mench and Fargue, 1994). The amino acids secreted by the roots of ryegrass reduce the pH value of rhizosphere soil and make the organic Zn of rhizosphere soil higher than that of non-rhizosphere soil (Xu et al., 2007).

Zn supply is a direct and effective method to solve the low availability of Zn in soil (Almendros et al., 2013; Guo et al., 2015). Furthermore, nitrogen (N) application or N combined with Zn application is beneficial for increasing Zn concentration, enhancing Zn translocation to grain in wheat, especially when wheat is cultured in Zn-deficient soil (Kutman et al., 2010; Li et al., 2015). N combined with Zn application can enhance Zn uptake via membrane transport and stimulate root development (Nie et al., 2017). When Zn has been absorbed, N in combination with Zn facilitates translocation of Zn to grain (Zhao et al., 2016). However, little is known about how N combined with Zn application affects the Zn fractions or their availability in soil to increase the absorption of Zn, especially through the root system of plants.

The aims of this study were: (i) to re-examine the effect of N combined with Zn application on Zn concentration in winter wheat; (ii) to investigate available Zn, pH, and Zn chemical fractions in soil influenced by the root system of winter wheat; and (iii) to investigate available Zn, pH, and Zn chemical fractions in soil in response to different N application rates, combined with Zn during pot trials. The results are expected to provide a possible reason for the Zn absorption enhanced by N combined with Zn application from the change in Zn availability in soil.

## Materials and Methods

### Plant Growth Description

Winter wheat (*Triticum aestivum* cv. Yunong 202) seeds were sown in ceramic bowls (inner diameter 28 cm and height 20 cm) each filled with 8 kg of Zn-deficient calcareous soil. The ceramic bowls were undrained to avoid the nutrients loss. The physical and chemical properties of soil were as follows: pH 7.34 (soil: water ratio of 1:2.5), organic matter 11.8 g kg^-1^, alkaline hydrolysis N 78.4 mg kg^-1^, Olsen-P 11.0 mg kg^-1^, available K 164 mg kg^-1^, and DTPA-extractable Zn 1.0 mg kg^-1^. The experiment was conducted at the Science Park of Henan Agricultural University (N 34°52′, E 113°35′), Zhengzhou City, Henan Province, China.

### Experimental Design

Two rates of Zn (0 and 10 mg kg^-1^ soil) and three rates of N [0.05 (low), 0.2 (medium), and 0.4 (high) g kg^-1^ soil] were applied by using ZnSO_4_⋅7H_2_O and Ca(NO_3_)_2_⋅2H_2_O, respectively. Low N and medium N pots were supplemented with CaCl_2_⋅2H_2_O to ensure the same extra amount of Ca for all treatments. Six treatments were applied including N_0.05_Zn_0_, N_0.2_Zn_0_, N_0.4_Zn_0_, N_0.05_Zn_10_, N_0.2_Zn_10_, and N_0.4_Zn_10_. Control (no plant) pots were used to compare with the N and Zn treatments. Each treatment was replicated three times. To meet the P and K requirements, KH_2_PO_4_ and KCl were applied in each pot at the rates of 0.15 g P_2_O_5_ kg^-1^ soil and 0.20 g K_2_O kg^-1^ soil. Before sowing, all the fertilizers including half of N for each N treatment were mixed into a solution with deionized water and supplied into soil, and the remaining N was split into two equal applications in the soil at the jointing and booting stages. One mL of Arnon nutrient solution (non-Zn) per kg soil was supplemented to each bowl to avoid trace element deficiency, including Fe-EDTA 0.025 mg, MnCl_2_⋅H_2_O 1.81 mg, CuSO_4_⋅5H_2_O 0.08 mg, H_3_BO_3_ 2.86 mg, and (NH_4_)_6_Mo_7_O_20_ 0.02 mg kg^-1^ soil. All the chemical reagents used were of analytical grade, and deionized water was used during the experimental period.

Twenty seeds were sown in each pot on October 17, 2015, and ten seedlings were retained in each pot after thinning. The shoots of winter wheat and soils with and without plants were sampled at the tillering (85 days, Zadoks stage: 23), jointing (168 days, Zadoks stage: 36), grain filling (192 days, Zadoks stage: 77) and mature (216 days, Zadoks stage: 99) stages, as well as grain at the mature stage. The shoots and grain of plants were oven-dried at 65°C and analyzed for elemental concentrations. The soil was air dried, ground, passed through a 1-mm mesh sieve, and then thoroughly mixed.

### Zn and N Analysis

According to the method of Bao (2002), Zn and N concentrations were determined using atomic absorption spectrometry (AAS) and Kjeldahl method, respectively. For the Zn analysis, five mL of the mixture HNO_3_:HClO_4_ (4:1, v/v) was used to digest the dry samples, and the Zn concentration of the digested samples was determined via flame AAS (ZEEnit 700, Analytik Jena AG, Germany). For the N analysis, five mL of H_2_SO_4_ and six drops of H_2_O_2_ were used to digest the dry samples, and the total N concentration of the digested samples was determined using a nitrogen autoanalyzer (BRAN LUEBBE AA3 Autoanalyzer, Germany). The certified standard reference materials (bush leaves, GBW07602 (GSV-1)), purchased from the National Center of Standard Material in China, was used to check the measurements.

### Soil Chemical Properties Analysis

The physical and chemical properties of the soil including pH, organic matter, alkaline hydrolysis N, Olsen-P, available K and DTPA-extractable Zn were analyzed following the methods reported by Bao (2002).

### Chemical Fractions of Zn in Soil

The method of sequential extraction used in this experiment was selected from the procedure of Tessier et al. (1979) and the revised method by Jiang et al. (1990). Six chemical fractions were separated as exchangeable, loose organic-bound, carbonate-bound, Fe-Mn oxides-bound, tight organic-bound, and residual fractions. A summary of the procedure is as follows:

Two grams of soil was weighed into a 50-mL polycarbonate centrifuge tube and added to the following solution to extract the different fractions. (1) Exchangeable: soil was extracted with 20 mL of 1 mol L^-1^ Mg(NO_3_)_2_ (pH 7.0) for 2 h at 25°C with continuous shaking. (2) Loose organic: residue from the exchangeable fraction was extracted with 20 mL of 0.1 mol L^-1^ Na_4_P_2_O_7_ (pH 9.5) for 2 h at 25°C with continuous shaking. (3) Carbonate: residue from the loose organic fraction was extracted with 20 mL of 1 mol L^-1^NaOAc (adjusted to pH 5.0 with HOAc) for 5 h at 25°C with continuous shaking. (4) Fe-Mn oxides: residue from the carbonate fraction was extracted with 20 mL of 0.1 mol L^-1^ NH_2_OH⋅HCl (pH 7.0) for 0.5 h at 25°C with continuous shaking. (5) Tight organic: residue from the Fe-Mn oxides fraction was extracted with 20 mL of 30% H_2_O_2_ (pH 2.0) for 2 h at 85°C with continuous shaking. A second 20 mL of 30% H_2_O_2_ (pH 2.0) was added and heated again at 85°C for 3 h with intermittent shaking. After cooling, 20 mL of 1 mol L^-1^ Mg(NO_3_)_2_ (pH 7.0) was added, and the samples were shaken for 2 h at 25°C. (6) Residual: two grams of soil was weighed into a 50-mL Teflon crucible and digested using HCl-HNO_3_-HClO_4_-HF, which was used to analyze the total Zn concentration. The residual Zn was obtained via subtracting the sum of exchangeable, loose organic-bound, carbonate-bound, Fe-Mn oxides-bound, and tight organic-bound Zn concentrations from the total Zn concentration.

The Zn concentration of each extracted sample was determined via flame AAS (ZEEnit 700, Analytik Jena AG, Germany).

### Calculations and Statistical Analysis

The chemical fractions of Zn were calculated as a percentage of the total Zn in all fractions.

The significance of the treatment effects and their interactions on the reported traits were evaluated by two-way or three-way ANOVA. The data are presented as averages of three replicates. Significant differences between means were determined using least significant difference (LSD) multiple comparisons (*P* < 0.05).

## Results

### Yield and Yield Components

Two-way ANOVA revealed significant interactive effects of N and Zn application on the spike number per pot, the thousand kernel weight and the grain yield in winter wheat (**Supplementary Table S1**). There were significant effects of N application on the spike number per pot, the grain number per spike and the grain yield, as well as significant effects of Zn application on the spike number per pot, the thousand kernel weight and the grain yield in winter wheat.

Compared with N_0.05_ treatment, N_0.2_ treatment significantly increased the spike number per pot at each Zn treatment and the grain number per spike at Zn0 treatment; N_0.4_ treatment significantly increased the spike number per pot and the grain yield at each Zn treatment (**Table 1**). Zn_10_ treatment significantly increased the spike number per pot and the grain yield only with N_0.4_ treatment. The spike number per pot, the thousand kernel weight and the grain yield were greater with N_0.4_Zn_10_ treatment than with any other treatment, but the highest value of grain number per spike was observed with the N_0.2_Zn_10_ treatment.

**Table 1:** Yield and yield components of winter wheat (*Triticum aestivum* cv Yunong 202), grown at 0.05, 0.2, and 0.4 g N kg^-1^ soil in a pot with 0, and 10 mg Zn kg^-1^ soil supply for mature.

N supply	Spike number per pot		Grain number per spike		Thousand kernel weight		Grain yield (g pot^-1^)	
	Zn_0_	Zn_10_	Zn_0_	Zn_10_	Zn_0_	Zn_10_	Zn_0_	Zn_10_
N_0.05_	10.7d	13.0d	32.1b	35.5ab	48.5ab	48.5ab	17.6c	21.1bc
N_0.2_	18.3c	20.3c	43.6a	43.8a	43.7b	46.6b	22.2bc	25.0b
N_0.4_	24.0b	39.7a	34.9ab	41.9a	48.5ab	56.5a	24.4b	38.1a


*Values are means of three independent replicates. For each trait, means followed by different letters are significantly different from each other according to two-way ANOVA followed by least significant difference (LSD) multiple comparison (*P* < 0.05).*

### N and Zn Concentration in Shoots and Grain

Two-way ANOVA revealed significant interactive effects of N and Zn application on the N concentration in shoots and grain at the tillering, jointing and mature stages and on the Zn concentration in shoots and grain at each growth stage (**Supplementary Table S1**). N and Zn application had significant effects on N and Zn concentrations in shoots and grain at different growth stages, except for the N concentration in shoots at the mature stage.

N and Zn concentrations in shoots and grain were increased with the increase in N application at each growth stage, at each Zn application rate (**Tables 2**, **3**). At the tillering stage, Zn application significantly increased N and Zn concentrations in shoots at different N application rates; at the jointing stage, Zn application significantly increased shoot N concentration with N_0.2_ treatment and shoot Zn concentration withN_0.2_ and N_0.4_ treatments; at the grain filling stage, Zn application significantly increased shoot N concentration with N_0.2_ treatment and shoot Zn concentration with N_0.4_ treatment; at the mature stage, Zn application significantly increased N and Zn concentrations in shoots with N_0.4_ treatment and Zn concentration in shoots with N_0.05_ treatment, as well as grain N concentration at each N treatment and grain Zn concentration with N_0.2_ and N_0.4_ treatments. N and Zn concentrations in the shoots and grain of winter wheat were highest with N_0.4_Zn_10_ treatment compared with the other treatments.

**Table 2:** N and Zn concentration in shoot of winter wheat (*Triticum aestivum* cv Yunong 202), grown at 0.05, 0.2, and 0.4 g N kg^-1^ soil in a pot with 0, and 10 mg Zn kg^-1^ soil supply at different growth stage.

Growth Stage	N supply	N concentration (g/kg)		Zn concentration (mg/kg)	
		Zn_0_	Zn_10_	Zn_0_	Zn_10_
Tillering	N_0.05_	5.41f	7.28e	8.70f	11.7e
	N_0.2_	9.33d	10.6c	14.3d	19.6c
	N_0.4_	18.1b	19.6a	25.9b	39.0a
Jointing	N_0.05_	17.9d	15.9cd	28.1e	31.1e
	N_0.2_	22.2c	31.9b	45.4d	74.9b
	N_0.4_	36.2ab	38.3a	59.8c	91.6a
Grain filling	N_0.05_	3.92c	4.20c	10.7d	12.9cd
	N_0.2_	5.46c	9.89b	14.8bc	12.9cd
	N_0.4_	13.8a	14.6a	17.0b	25.9a
Mature	N_0.05_	5.04d	4.38d	5.63e	10.1d
	N_0.2_	6.91c	7.65bc	13.2cd	16.2c
	N_0.4_	8.96b	11.8a	23.2b	43.3a


*Values are means of three independent replicates. For each trait, means followed by different letters are significantly different from each other according to two-way ANOVA followed by least significant difference (LSD) multiple comparison (*P* < 0.05)*.

**Table 3:** N and Zn concentration in grain of winter wheat (*Triticum aestivum* cv. Yunong 202), grown at 0.05, 0.2, and 0.4 g N kg^-1^ soil in a pot with 0, and 10 mg Zn kg^-1^ soil supply for mature.

N supply	N concentration (g/kg)		Zn concentration (mg/kg)	
	Zn_0_	Zn_10_	Zn_0_	Zn_10_
N_0.05_	19.0d	20.9c	36.8c	43.7c
N_0.2_	23.5b	22.0c	36.8c	56.4b
N_0.4_	24.6b	27.4a	66.0b	102a


*Values are means of three independent replicates. For each trait, means followed by different letters are significantly different from each other according to two-way ANOVA followed by least significant difference (LSD) multiple comparison (*P* < 0.05).*

### Available Zn Concentration in Soil

As revealed by three-way ANOVA, the available Zn concentration in soil was significantly affected by the plant, the N and Zn applications, and the interaction of plant × N, plant × Zn, N × Zn, and plant × N × Zn at each growth stage, except for the plant × Zn interaction at the grain filling stage (**Supplementary Table S2**).

**FIGURE 1:**
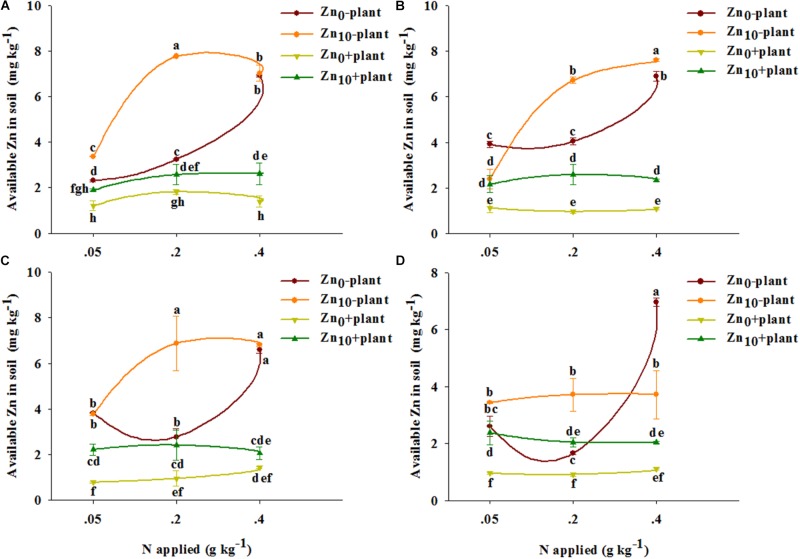
Available Zn concentration in soil of winter wheat (*Triticum aestivum* cv Yunong 202) at the tillering **(A)**, jointing **(B)**, grain filling **(C)**, and mature **(D)** stage with three N application rates (0.05, 0.2, and 0.4 g N kg^-1^ soil) and two Zn application rates (0 and 10 mg Zn kg^-1^ soil), without and with plants. Values are means of three independent replicates. Error bars represent 1 SE. Means followed by different letters are significantly different from each other according to three-way ANOVA followed by least significant difference (LSD) multiple comparison (*P* < 0.05).

With each treatment and at each growth stage, the available Zn concentration in soil without plants was higher than that in soil with plants (**Figure 1**).

For the soil without plants, N_0.2_ treatment significantly increased the available Zn concentration with Zn_0_ and Zn_10_ treatments at the tillering stage and only increased the concentration with Zn_10_ treatment at the jointing and grain filling stages; N_0.4_ treatment significantly increased the available Zn concentration with each Zn treatment at different growth stages, except for treatment with Zn_10_ at the mature stage (**Figure 1**). The available Zn concentration was significantly increased by Zn application with the treatment of N_0.05_ and N_0.2_ at the tillering stage, with the treatment of N_0.2_ and N_0.4_ at the jointing stage, and with N_0.2_ treatment at the grain filling and mature stages; however, Zn application significantly decreased the available Zn concentration with N_0.05_ treatment at the jointing stage and with N_0.4_ treatment at the mature stage.

For the soil with plants, N_0.2_ and N_0.4_ treatment significantly increased the available Zn concentration with Zn_10_ treatment at the tillering stage, and N_0.4_ treatment significantly increased the available Zn concentration with Zn_0_ treatment at the grain filling stage (**Figure 1**). The available Zn concentration was significantly increased by Zn application for each N treatment at different growth stages, except for that with N_0.05_ treatment at the tillering stage and with N_0.4_ treatment at the grain filling and mature stages.

Available Zn concentrations in soil with and without plants were highest with N_0.2_Zn_10_ or N_0.4_Zn_10_ treatment compared with the other treatments at the tillering, jointing and grain filling stages.

### pH in Soil

As revealed by three-way ANOVA, the pH in soil was significantly affected by plants, by N application, and by the plant × N interaction at each growth stage (**Supplementary Table S2**). Zn application had significant effects on soil pH at the tillering, grain filling and mature stages. The interaction of plant × Zn significantly affected soil pH at the grain filling and mature stages. The interaction of N × Zn significantly affected soil pH at the tillering, grain filling and mature stages. The interaction of plant × N × Zn significantly affected soil pH at the tillering, jointing and grain filling stages.

**FIGURE 2:**
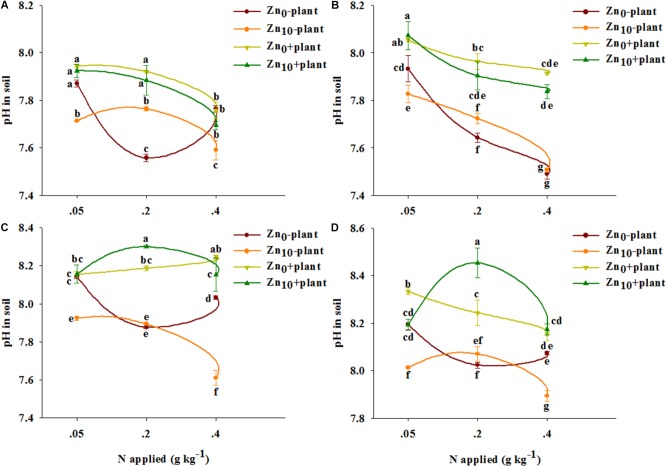
pH in soil of winter wheat (*Triticum aestivum* cv. Yunong 202) at the tillering **(A)**, jointing **(B)**, grain filling **(C)**, and mature **(D)** stage with three N application rates (0.05, 0.2, and 0.4 g N kg^-1^ soil) and two Zn application rates (0 and 10 mg Zn kg^-1^ soil), without and with plants. Values are means of three independent replicates. Error bars represent 1 SE. Means followed by different letters are significantly different from each other according to three-way ANOVA followed by least significant difference (LSD) multiple comparison (*P* < 0.05).

For each treatment and each growth stage, pH in soil without plants was lower than that in soil with plants, except for that with N_0.05_Zn_0_ treatment at the tillering and grain filling stages (**Figure 2**).

For the soil without plants, N_0.2_ treatment significantly decreased pH with Zn_0_ treatment at the tillering, grain filling and mature stages, and decreased it with Zn_0_ and Zn_10_ treatment at the jointing stage; N_0.4_ treatment significantly decreased pH with each Zn treatment at different growth stages (**Figure 2**). pH was significantly decreased by Zn application with N_0.05_ treatment at each growth stage; for N_0.2_ treatment, pH was significantly decreased by Zn application at the tillering stage; Zn application significantly decreased pH with N_0.4_ treatment at the tillering, grain filling and mature stages.

For the soil with plants, N_0.2_ treatment significantly decreased pH with Zn_10_ treatments at the jointing stage, as well as with Zn_0_ treatment at the mature stage, but increased pH with Zn_10_ treatment at the grain filling and mature stages; N_0.4_ treatment significantly decreased pH with Zn_0_ and Zn_10_ treatments at the tillering and jointing stages, as well as with Zn_0_ treatment at the mature stage, but increased pH with Zn_0_ treatment at the grain filling stage (**Figure 2**). Zn application significantly increased pH with N_0.2_ treatment but decreased it with N_0.4_ treatment at the grain filling stage; Zn application significantly decreased pH with N_0.05_ treatment but increased it with N_0.4_ treatment at the mature stage.

pH in soil with and without plants was highest with N_0.4_Zn_10_ treatment compared with the other treatments.

### Chemical Fractions of Zn Concentration in Soil

Three-way ANOVA revealed significant interaction of plant × N × Zn on the exchangeable and residual Zn concentration at the tillering stage, the loose organic-bound Zn concentration at each growth stage, the carbonate-bound Zn concentration at the tillering and mature stages, the Fe-Mn oxides-bound Zn concentration at the jointing and grain filling stages, and the tight organic-bound Zn concentration at the jointing, grain filling and mature stages (**Supplementary Table S3**).

With the treatments of N_0.2_Zn_10_ and N_0.4_Zn_10_ at different growth stage, the exchangeable Zn in soil without plants was lower, but the carbonate-bound, Fe-Mn oxides-bound and residual Zn in soil without plants was higher than that in soil with plants (**Table 4**). There were opposite results for loose organic- and tight organic-bound Zn with and without plants in different treatments at various growth stages.

**Table 4:** Zn fractions in soil of winter wheat (*Triticum aestivum* cv. Yunong 202), grown at 0.05, 0.2, and 0.4 g N kg^-1^ soil in a pot with 0, and 10 mg Zn kg^-1^ soil supply at different growth stage (mg kg^-1^).

Fractions	Plant	N supply	Growth Stage							
			Tillering		Jointing		Grain filling		Matureg	
			Zn_0_	Zn_10_	Zn_0_	Zn_10_	Zn_0_	Zn_10_	Zn_0_	Zn_10_
Exchangeable	No plant	N_0.05_	0.87a	0.68b	0.80a	0.64b	1.29ab	1.36a	0.58ab	0.40bc
		N_0.2_	0.41e	0.49de	0.45c	0.38c	0.42d	0.43d	0.27c	0.31c
		N_0.4_	0.58cd	0.50de	0.23d	0.43c	0.46d	0.57d	0.46bc	0.34c
	plant	N_0.05_	0.48de	0.66bc	0.40c	0.41c	1.13c	1.16bc	0.29c	0.27c
		N_0.2_	0.64bc	0.81a	0.60b	0.59b	1.12c	1.34a	0.65a	0.56ab
		N_0.4_	0.66bc	0.69b	0.47c	0.58b	1.24abc	1.41a	0.42bc	0.55ab
Loose organic-bound	No plant	N_0.05_	1.39gh	1.29h	3.01cde	3.54bcd	4.03c	2.49ef	1.40cd	0.77de
		N_0.2_	0.75h	0.91h	6.70a	3.09cde	3.34cde	3.53cd	0.91de	0.71e
		N_0.4_	8.64a	5.55b	3.16cde	2.05fg	8.05a	5.95b	0.78de	0.82de
	plant	N_0.05_	2.54ef	3.05de	1.73g	2.32efg	2.33f	3.63cd	1.96c	3.95a
		N_0.2_	2.17f	3.53cd	2.74def	3.79bc	2.00f	1.91f	2.73b	3.20b
		N_0.4_	1.97fg	3.89c	2.05fg	4.23b	2.75def	2.56ef	0.90de	1.12de
Carbonate-bound	No plant	N_0.05_	2.31ef	2.83de	3.41bc	3.92ab	1.81abc	1.65bcd	3.87ab	3.88ab
		N_0.2_	2.41ef	4.49a	2.67de	3.99ab	1.23e	1.95ab	3.73b	4.11ab
		N_0.4_	3.98ab	3.91ab	3.01cd	4.21a	1.87abc	1.87abc	4.63a	3.38b
	plant	N_0.05_	2.33ef	3.39bcd	2.28e	2.31e	1.18e	1.49cde	1.51de	1.69cde
		N_0.2_	2.11f	3.47bc	2.59de	2.21e	1.30de	1.66bcd	1.22e	2.35c
		N_0.4_	2.19f	3.18cd	2.72de	3.19cd	1.58bcde	2.08a	1.34e	2.28cd
			Zn_0_	Zn_10_	Zn_0_	Zn_10_	Zn_0_	Zn_10_	Zn_0_	Zn_10_
Fe-Mn oxides-bound	No plant	N_0.05_	0.48abc	0.56a	0.65de	0.66d	0.51c	0.67abc	0.48e	0.51de
		N_0.2_	0.41abcde	0.51ab	0.64de	0.69cd	0.61bc	0.68ab	0.52cde	0.67abc
		N_0.4_	0.52ab	0.45abcd	0.75cd	0.80c	0.79a	0.17e	0.69ab	0.75a
	plant	N_0.05_	0.33cde	0.28e	1.06b	1.27a	0.53bc	0.64abc	0.54bcde	0.53cde
		N_0.2_	0.32de	0.45abcd	1.05b	0.53ef	0.57bc	0.53bc	0.65abcd	0.64abcd
		N_0.4_	0.39bcde	0.41abcde	0.52ef	0.50f	0.27de	0.35d	0.67abc	0.47e
	No plant	N_0.05_	0.62c	0.77abc	0.72abc	0.46e	0.33e	0.83a	1.20ab	1.25a
Tight organic-bound		N_0.2_	0.78ab	0.80a	0.81a	0.29f	0.71c	0.72c	1.10abcd	0.93cde
		N_0.4_	0.74abc	0.69abc	0.49e	0.66bcd	0.75bc	0.79ab	0.98cde	1.13abc
	plant	N_0.05_	0.63bc	0.69abc	0.70abcd	0.59cde	0.13f	0.14f	1.28a	0.70f
		N_0.2_	0.63bc	0.68abc	0.56de	0.46e	0.16f	0.17f	0.68f	1.03bcde
		N_0.4_	0.65abc	0.77abc	0.68abcd	0.79ab	0.28e	0.46d	0.85ef	0.93de
Residual	No plant	N_0.05_	74.6ab	78.9a	38.6e	52.5bcd	78.4bcd	83.2b	79.2bc	83.5ab
		N_0.2_	44.6d	64.4bc	51.9bcd	54.5abc	92.7b	89.8b	72.8cd	81.7abc
		N_0.4_	53.1d	51.1d	52.0bcd	58.9ab	87.9b	119a	89.6a	82.5abc
	plant	N_0.05_	45.2d	43.9d	46.7cd	59.3ab	56.5ef	53.4f	48.3g	60.5ef
		N_0.2_	45.4d	51.7d	46.4de	58.9ab	54.2ef	64.4def	58.7f	63.4def
		N_0.4_	49.3d	64.1c	52.5bcd	61.3a	68.1cde	80.1bc	56.3fg	68.7de


*Values are means of three independent replicates. For each trait, means followed by different letters are significantly different from each other according to three-way ANOVA followed by least significant difference (LSD) multiple comparison (*P* < 0.05).*

N_0.2_ and N_0.4_ treatments significantly decreased the exchangeable Zn concentration in soil without plants, but increased that in soil with plants at each growth stage, regardless of Zn application (**Table 4**). N_0.2_ or N_0.4_ treatment significantly increased the loose organic-bound Zn concentration in soil without plants, with Zn_0_ and Zn_10_ treatments at various growth stages; N_0.2_ and N_0.4_ treatment significantly increased the loose organic-bound Zn concentration in soil with plants at the tillering and jointing stage, but decreased that at the grain filling and mature stage with Zn_10_ treatment. The carbonate-bound Zn concentration in soil without and with plants was significantly increased by N_0.2_ or N_0.4_ treatment with Zn_10_ at each growth stage. N_0.4_ treatment significantly increased the Fe-Mn oxides-bound Zn concentration in soil without plant with Zn_0_ and Zn_10_ treatment at the tillering, jointing and mature stages, but decreased that in soil with plant with Zn_0_ and Zn_10_ treatment at various growth stages. There were different results for tight organic-bound Zn in soil with and without plants influenced by N application rates in different Zn application rates at various growth stages. N_0.2_ and N_0.4_ treatment significantly decreased the residual Zn concentration in soil without plants at the tillering stage, but increased that in soil without plants at the jointing, grain filling and mature stage and that in soil with plants at each growth stage, regardless of Zn application. Zn application had different influence on the each fraction of Zn in soil without and with plants with different N treatment at various growth stages.

N_0.2_Zn_10_ or N_0.4_Zn_10_ treatment had lower exchangeable and loose organic-bound Zn but higher carbonate- and Fe-Mn oxides-bound and residual Zn concentrations in soil without plants. The exchangeable, loose organic-, carbonate-bound, tight organic-bound and residual Zn concentrations in soil with plants were higher with N_0.2_Zn_10_ or N_0.4_Zn_10_ treatment compared with the other treatments.

### Proportions of Zn Chemical Fractions in Soil

Three-way ANOVA revealed significant interaction of plant × N × Zn on the exchangeable Zn proportion at the tillering and jointing stages, the loose organic-bound Zn proportion at each growth stage, the carbonate- and Fe-Mn oxides-bound Zn proportion at the jointing and grain filling stages, the tight organic-bound Zn proportion at the jointing, grain filling and mature stages, and the residual Zn proportion at the grain filling stages (**Supplementary Table S4**).

**FIGURE 3:**
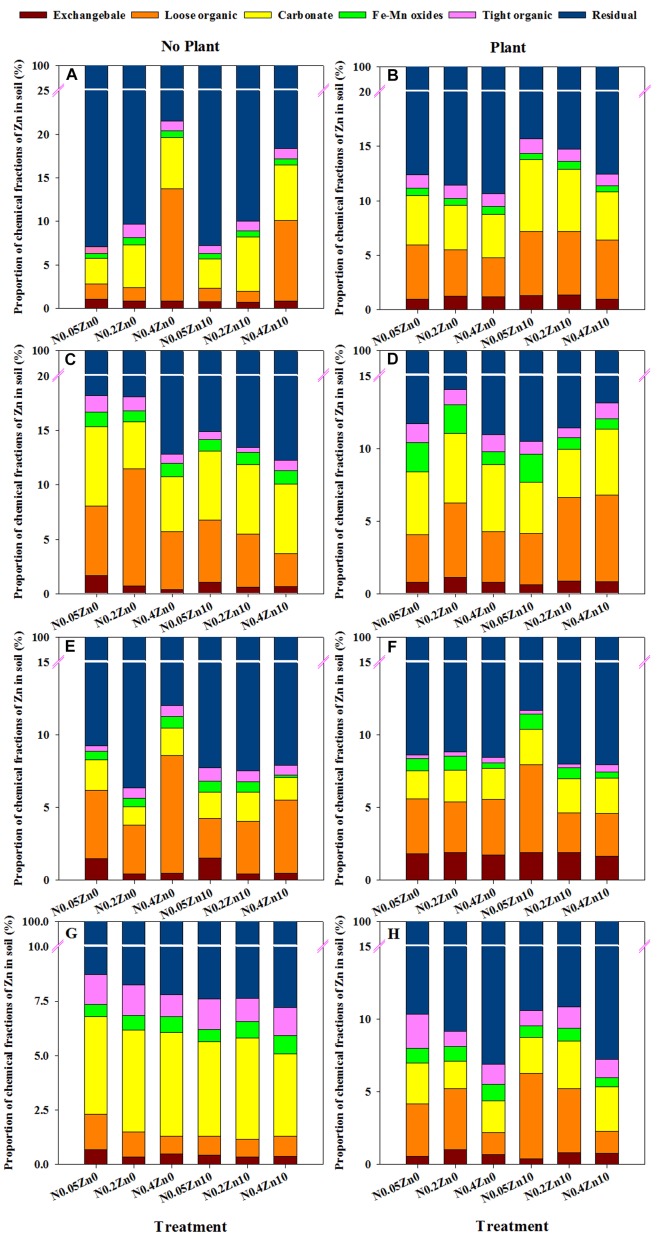
Proportion of chemical fractions of Zn in soil no-growing **(A,C,E,G)** and growing **(B,D,F,H)** winter wheat (*Triticum aestivum* cv Yunong 202) at the tillering **(A,B)**, jointing **(C,D)**, grain filling **(E,F)** and mature **(G,H)** stage with three N application rates (0.05, 0.2, and 0.4 g N kg^-1^ soil) and two Zn application rates (0 and 10 mg Zn kg^-1^ soil), without and with plants. The proportion of chemical fractions of Zn (%) was calculated as the percentage of Zn concentrations in each fraction to all fractions. Values are means of three independent replicates.

The differences for each fraction of Zn proportion between soil without and with plants was depended on the different treatment at various growth stages (**Figure 3**). With Zn_0_, N_0.2_ and N_0.4_ treatments decreased the proportion of exchangeable and tight organic-bound Zn in soil without plants but increased the proportion of exchangeable and residual Zn in soil with plants at various growth stages; there were opposite results in the proportion of loose organic-, carbonate-, Fe-Mn oxides-, tight organic-bound and residual Zn in soil without and with plants influenced by N_0.2_ and N_0.4_ treatments at different growth stages. With Zn_10_, N_0.2_ treatment decreased the proportion of exchangeable Zn in soil without plants but increased that in soil with plants; N_0.4_ treatment decreased the proportion of exchangeable Zn in soil without plants at various growth stages; different results were observed in the proportion of Zn fractions in soil without and with plants influenced by N_0.2_ and N_0.4_ treatments at different growth stages. Zn application also had different influence on the proportion of each Zn fraction in soil without and with plants with different N treatment at various growth stages.

## Discussion

### N Combined With Zn Application Increased Zn Concentration in Winter Wheat

The grain yield, the yield components and the concentrations of Zn and N in the shoots of winter wheat were all significantly increased by N combined with Zn application, which produced the highest value (**Tables 1**, **2**). These results suggested that N combined with Zn application was more beneficial to enhance the growth and that this combination increased Zn and N concentrations in shoots compared to N or Zn application alone. The enhancement of Zn concentration following the application of N combined with Zn has been suggested for the enhanced growth of plants (Aciksoz et al., 2011). Our results suggested that the increased Zn concentration in shoots might be partially related to the enhanced growth that occurred following the application of N combined with Zn. On the other hand, N application had a positive effect on Zn absorption and root-to-shoot transport as Zn was supplied (Erenoglu et al., 2011). N combined with Zn application significantly increased Zn concentration in the shoot and grain of winter wheat (**Tables 2**, **3**), suggesting Zn absorption and transfer from roots to shoots and translocation to grain, consistent with earlier studies with maize (Mehdi et al., 2012; Jamil et al., 2015). Zhao et al. (2016) showed that N combined with Zn application was beneficial for uptake, transfer and accumulation in winter wheat. Xue et al. (2012) and Nie et al. (2017) also observed that there was a significant positive correlation between Zn and N concentration and revealed that the enhanced Zn membrane transport and stimulated root development by N combined with Zn application in winter wheat was one reason for increasing Zn absorption and transfer to shoots. Thus, to increase Zn concentration in wheat grain cultured in Zn-deficient soil, a combination of high N with Zn application is a better method.

### N Combined With Zn Application Increased Available Zn Concentration by Decreasing pH in Soil

Calcareous soil is widely distributed in the Northern Winter Wheat Region of China. The soil used in our study was calcareous soil, and its available Zn (DTPA-Zn) concentration was 1.02 mg kg^-1^, which was in the range of potential Zn deficiency in soil (Liu, 1994). The potentially Zn deficient soil was selected in our study based on the following three reasons. Firstly, the soils in most area of Henan Province are potentially Zn-deficient (Sun and Cai, 1984). Secondly, as the main grain producing area, Henan Province has a very high yield of grain crops, including winter wheat. The production of some high-yield or super-high-yield crop varieties result in the increase in the demand for Zn nutrition of crops, thus Zn uptake in crops from the soil is also high. Thirdly, in our previous studies, this potentially Zn deficient soil was used as experimental material and found that the application of Zn fertilizer had a significant effect on increasing grain yield of winter wheat (Liu et al., 2015; Zhao et al., 2016; Nie et al., 2017). Calcareous soil is characterized by a high CaCO_3_ concentration, a high pH value and a low organic matter concentration, which can enhance the Zn fixation ability of soil and can reduce the bioavailability of Zn, thus inhibiting Zn uptake in plant roots (Alloway, 2008). In our study, high N (N_0.4_) application induced significant increases in available Zn concentration in soil without plants in the absence of Zn application at each growth stage; medium N (N_0.2_) application only increased it at 85 days (**Figure 1**). This suggested that the available Zn concentration was increased only when N was supplied at high levels under Zn-deficient conditions. With Zn application, N_0.2_ and N_0.4_ increased the Zn concentration in soil without plants at 85, 168, and 192 days (**Figures 1A–C**), indicating that N combined with Zn application could increase the availability of Zn in soil without plants. According to our conversion, N supplied at 225 and 450 kg ha^-1^ and Zn supplied at 11 kg ha^-1^ might be beneficial for the increase of Zn availability in soil under the field condition. Our previous study showed that N (180 and 270 kg ha^-1^) combined with Zn (15 and 30 kg Zn ha^-1^) treatment enhanced Zn absorption and accumulation in winter wheat grown in Zn-deficient soil (Zhao et al., 2016), which might be the increased Zn availability in soil. Meanwhile, N combined with Zn application could increase the availability of Zn in soil with plants, which might be one reason for enhanced Zn absorption in winter wheat by application of N combined with Zn. Our results are consistent with those of Lu et al. (2010), who noted that N combined with Zn application induced larger concentrations of available Zn in the 0–20 cm and 20–40 cm layers than with N and Zn application alone. Our results also showed that winter wheat roots significantly decreased the available Zn concentration in soil with plants compared with soil without plants at each growth stage (**Figure 1**). The major reason for this difference in available Zn concentration could be the Zn uptake by the roots of winter wheat (Mengel and Kirby, 1987).

The availability of Zn in soil increases with decreasing pH because the decreased pH weakens the affinity of soil to Zn by influencing Zn hydroxylation or the soil adsorption surface (Msaky and Calvet, 1990; Pardo and Guadalix, 1996). Our results showed that both N_0.2_ and N_0.4_ treatment decreased pH in Zn-deficient soil without plants, and only N_0.4_ treatment decreased pH under Zn sufficiency at different growth days (**Figure 2**). Nitrate (NO_3_^-^) was used in our study as the N source, thus a possible reason for decreased pH in soil by N application was related to the leaching of NO_3_^-^ inducing permanent soil acidity when the loss of NO_3_^-^ uncouples a hydrogen ion (H^+^)-balancing system (Bolan et al., 1991). In addition, after transformation of NO_3_^-^ to ammonium ion (NH_4_^+^) in soil, H^+^ is released through nitrification of NH_4_^+^ (Zhou et al., 2014). For soils with plants, apart from the generation of H^+^ in the soil N cycle (such as NO_3_^-^ leaching and NH_4_^+^ nitrification), the change in pH is influenced by two kinds of root activities: root excretion, such as organic acid, can reduce the pH value, and plants absorb or secrete H^+^ to maintain a neutral environment on the root-soil interface while absorbing the nutrient ions (Van Beusichem et al., 1988; Paterson et al., 2006). H^+^ can enter the root cells through the H^+^/NO_3_^-^ co-transfer system (Britto and Kronzucker, 2006), and the uptake of NO_3_^-^ into plants has led to H^+^ uptake and consequently to increasing pH in soil. We observed that the pH in soil with plants was higher than in soil without plants (**Figure 2**), suggesting that the increased pH by roots was caused by the synergetic absorption of H^+^/NO_3_^-^ (Britto and Kronzucker, 2006). In our study, compared with N application alone, low N (N_0.05_) combined with Zn application decreased pH in soil with plants only at the mature stage (**Figure 2D**). This may have been because when N was supplied at low levels, the amount of H^+^ generated in the soil N cycle was equal to the uptake by winter wheat, and the H^+^ uptake declined with the decreasing uptake of NO_3_^-^ when wheat was mature; thus, the H^+^ generated in the N cycle of soil was dominant and decreased the pH. However, medium N (N_0.2_) combined with Zn application decreased pH at the tillering and jointing stages but increased it at the grain filling and mature stages (**Figure 2**). This suggests that the soil N cycle and root excretion were dominant when wheat was at the vegetative period, thus decreasing pH. In contrast, the synergetic absorption of H^+^/NO_3_^-^ was dominant when wheat was at the reproductive stage, which was related to the highest N uptake of wheat at the jointing and grain filling stages, thus increasing the pH (Delogua et al., 1998). High N (N_0.4_) combined with Zn application decreased the pH at different growth stages (**Figure 2**), indicating that the change in pH was more influenced by the soil N cycle or root excretion than by the synergetic absorption of H^+^/NO_3_^-^. Therefore, exploring which of the above mentioned processes influenced by different N application rates in changing the pH in soil with plants is worth further study. In brief, our results indicated that the combined influence of roots and the combination of N and Zn enhanced Zn availability via decreasing pH in soils, which was agree with the results of Chen et al. (2010).

### N Combined With Zn Application Enhanced Zn Availability by Affecting the Transformation and Distribution of Zn in Various Soil Fractions

Research has shown a close relationship between chemical fractions of Zn in soil and the availability of Zn in plants. Sungur et al. (2014) noted that plants can mainly take up Zn in the exchangeable and carbonate-bound fractions. Li et al. (2014) showed that exchangeable and organic matter-bound Zn in contaminated soil declined significantly after phytoextraction, which further indicates that the exchangeable and organic matter-bound fractions were more available to the plants than the other fractions. The availability of Zn in soil increased with the enhanced transformation of Zn from tight organic matter-bound to light organic matter-bound (Li et al., 2006). Guo et al. (2015) showed that the available Zn concentration was mainly influenced by exchangeable, light organic matter- and carbonate-bound fractions. In our experiment, N (N_0.2_ and N_0.4_) combined with Zn application decreased the exchangeable Zn but increased loose organic-, carbonate- and Fe-Mn oxides-bound Zn concentration in soil without plants at different growth days (**Table 4**), indicating that N combined with Zn application could influence the availability of Zn by changing the transformation between different fractions of Zn in soil. In addition, pH has been considered the most important factor affecting the transformation of Zn in various fractions (Wang et al., 2009). In soil with plants, N combined with Zn application increased the exchangeable, loose organic- and carbonate-bound Zn concentration in soil with plants at different growth stages (**Table 4**). This result also suggests that N combined with Zn application could increase the availability of Zn by enhancing the transformation of exchangeable, loose organic and carbonate-bound Zn from the other fractions. Possibly, root exudates provided exchangeable sites for Zn and increased cation exchange capacity in soil (Joergensen and Scheu, 1999; Khoshgoftarmanesh et al., 2018).

The highest proportion of Zn chemical fractions was the residual fraction (81.8–92.6% of total Zn), and a considerable amount of Zn was loose organic and carbonate-bound Zn at different growth stages (**Figure 3**), which agrees with the reports of Iyengar et al. (1981) and Lu et al. (2010). However, Xian (1987) reported that 30% of the total Zn was detected in the Fe-Mn oxides fraction. Xian (1989) showed that approximately 10% of the total Zn was associated with carbonates, and the proportion was raised according to the increase in inorganic C content in soil. Low, medium or high N combined with Zn application influenced the proportion of Zn in different fractions of soil with and without plants at different growth stages (**Figure 3**), also indicating that N combined with Zn application could influence the availability of Zn by changing the distribution of different Zn fractions in soil.

Winter wheat roots also significantly changed the concentration and proportion of Zn chemical fractions for soils with and without plants at each growth stage (**Table 4** and **Figure 3**). However, the change in Zn chemical fractions by roots depended on the N and Zn application rates. Khoshgoftarmanesh et al. (2018) noted that roots induced higher exchangeable and organic matter-bound Zn through the input of acidity and organic compounds by roots and the associated microbial activity. Li et al. (2014) showed that root uptake resulted in the largest decrease in the exchangeable and organic matter-bound Zn in contaminated soil. Xu et al. (2006) reported that planting rice could increase the concentration of carbonate- and Fe-Mn oxides-bound Zn in soil. Thus, roots activities also influenced the availability of Zn via changing the transformation between chemical fractions of Zn in soil. But whether or how the roots activities are influenced by N combined with Zn application in influencing the Zn fractions in soil is also worth further study.

## Conclusion

This study demonstrates that high N (0.4 g kg^-1^ N) combined with 10 mg kg^-1^ Zn application is beneficial for enhancing crop yields, Zn uptake and transport to grain in winter wheat. Under the action of winter wheat roots, a combination of 0.2 or 0.4 g kg^-1^ N and 10 mg kg^-1^ Zn can increase the Zn availability by decreasing the pH and enhancing the transformation and distribution of exchangeable, loose organic- and carbonate-bound Zn, thus promoting the Zn uptake in the roots of winter wheat.

## Author Contributions

ZN conceived and designed the experiments. PZ analyzed the data. SQ performed the experiments. HL wrote the paper.

## Conflict of Interest Statement

The authors declare that the research was conducted in the absence of any commercial or financial relationships that could be construed as a potential conflict of interest.
