# Supplementary material for: Chemical Fractions and Availability of Zinc in Winter Wheat Soil in Response to Nitrogen and Zinc Combinations
Source: Front Plant Sci. 2018 Oct 12;9:1489. doi: 10.3389/fpls.2018.01489 (PMC6194150; doi:10.3389/fpls.2018.01489)
Supplement: Supplementary file 1 [file Table_1.DOC]

**Supporting Information Tables S1–S4**

**Table S1** Two-way analysis of variance (ANOVA) of the effects of Zn and N application, as well as their interactions on the yield, yield components, and N and Zn concentrations, in shoots and grain of winter wheat (*Triticum aestivum* cv Yunong 202), grown at 0.05, 0.2, and 0.4 g N kg^-1^ soil in a pot with 0, and 10 mg Zn kg^-1^ soil supplied at different growth stages.

| Source of Variation (Treatment) |  | DF |  | Spike number per pot | | |  | Grain number per spike | | |  | Thousand kernel weight | | |
| --- | --- | --- | --- | --- | --- | --- | --- | --- | --- | --- | --- | --- | --- | --- |
|  |  |  |  | F |  | P |  | F |  | P |  | F |  | P |
| N application |  | 2 |  | 200 |  | <.001 |  | 5.49 |  | 0.02 |  | 3.23 |  | 0.08 |
| Zn application |  | 1 |  | 21.0 |  | <.001 |  | 0.31 |  | 0.59 |  | 5.30 |  | 0.04 |
| N×Zn |  | 2 |  | 52.1 |  | <.001 |  | 1.74 |  | 0.22 |  | 16.4 |  | <.001 |
| Source of Variation (Treatment) |  | DF |  | Grain yield | | |  | Shoot N concentration at tillering stage | | |  | Shoot N concentration at jointing stage | | |
|  |  |  |  | F |  | P |  | F |  | P |  | F |  | P |
| N application |  | 2 |  | 34.1 |  | <.001 |  | 518 |  | <.001 |  | 81.4 |  | <.001 |
| Zn application |  | 1 |  | 6.93 |  | 0.02 |  | 22.7 |  | <.001 |  | 6.07 |  | 0.03 |
| N×Zn |  | 2 |  | 4.08 |  | 0.04 |  | 0.26 |  | 0.78 |  | 7.03 |  | 0.01 |
| Source of Variation (Treatment) |  | DF |  | Shoot N concentration at grain filling stage | | |  | Shoot N concentration at mature stage | | |  | Shoot Zn concentration at tillering stage | | |
|  |  |  |  | F |  | P |  | F |  | P |  | F |  | P |
| N application |  | 2 |  | 64.3 |  | <.001 |  | 55.1 |  | <.001 |  | 547 |  | <.001 |
| Zn application |  | 1 |  | 6.12 |  | 0.03 |  | 4.78 |  | 0.27 |  | 163 |  | <.001 |
| N×Zn |  | 2 |  | 3.16 |  | 0.08 |  | 5.23 |  | <.001 |  | 29.5 |  | <.001 |
| Source of Variation (Treatment) |  | DF |  | Shoot Zn concentration at jointing stage | | |  | Shoot Zn concentration at grain filling stage | | |  | Shoot Zn concentration at mature stage | | |
|  |  |  |  | F |  | P |  | F |  | P |  | F |  | P |
| N application |  | 2 |  | 116 |  | <.001 |  | 48.9 |  | <.001 |  | 284 |  | <.001 |
| Zn application |  | 1 |  | 72.5 |  | <.001 |  | 13.3 |  | <.01 |  | 104 |  | <.001 |
| N×Zn |  | 2 |  | 13.4 |  | <.001 |  | 14.3 |  | <.001 |  | 37.2 |  | <.001 |
| Source of Variation (Treatment) |  | DF |  | Grain N concentration | | |  | Grain Zn Concentration | | |  |  | | |
|  |  |  |  | F |  | P |  | F |  | P |  |  |  |  |
| N application |  | 2 |  | 110 |  | <.001 |  | 81.3 |  | <.001 |  |  |  |  |
| Zn application |  | 1 |  | 9.74 |  | 0.01 |  | 47.5 |  | <.001 |  |  |  |  |
| N×Zn |  | 2 |  | 15.5 |  | <.001 |  | 7.83 |  | 0.01 |  |  |  |  |

**Table S2** Three-way analysis of variance (ANOVA) of the effects of plants, Zn and N application, as well as their interactions on the available Zn concentration and pH in soil of winter wheat (*Triticum aestivum* cv Yunong 202), with three N application rates (0.05, 0.2 and 0.4 g N kg^-1^ soil) and two Zn application rates (0 and 10 mg Zn kg^-1^ soil), without and with plants at different growth stages.

| Source of Variation (Treatment) |  | DF |  | Available Zn concentration at tillering stage | | |  | Available Zn concentration at jointing stage | | |  | Available Zn concentration at grain filling stage | | |
| --- | --- | --- | --- | --- | --- | --- | --- | --- | --- | --- | --- | --- | --- | --- |
|  |  |  |  | F |  | P |  | F |  | P |  | F |  | P |
| Plant |  | 1 |  | 534 |  | <.001 |  | 668 |  | <.001 |  | 194 |  | <.001 |
| N application |  | 2 |  | 97.8 |  | <.001 |  | 77.5 |  | <.001 |  | 13.7 |  | <.001 |
| Zn application |  | 1 |  | 102 |  | <.001 |  | 49.0 |  | <.001 |  | 27.2 |  | <.001 |
| Plant×N |  | 2 |  | 59.9 |  | <.001 |  | 71.7 |  | <.001 |  | 10.0 |  | <.001 |
| Plant×Zn |  | 1 |  | 13.3 |  | <.01 |  | 6.19 |  | 0.02 |  | 0.25 |  | 0.62 |
| N×Zn |  | 2 |  | 20.5 |  | <.001 |  | 25.1 |  | <.001 |  | 9.08 |  | <.01 |
| Plant×N×Zn |  | 2 |  | 27.4 |  | <.001 |  | 14.6 |  | <.001 |  | 6.20 |  | 0.01 |
| Source of Variation (Treatment) |  | DF |  | Available Zn concentration at mature stage | | |  | pH at tillering stage | | |  | pH at jointing stage | | |
|  |  |  |  | F |  | P |  | F |  | P |  | F |  | P |
| Plant |  | 1 |  | 115 |  | <.001 |  | 86.7 |  | <.001 |  | 177 |  | <.001 |
| N application |  | 2 |  | 18.3 |  | <.001 |  | 36.5 |  | <.001 |  | 66.2 |  | <.001 |
| Zn application |  | 1 |  | 7.02 |  | 0.01 |  | 6.89 |  | 0.01 |  | 1.08 |  | 0.31 |
| Plant×N |  | 2 |  | 17.7 |  | <.001 |  | 13.0 |  | <.001 |  | 7.81 |  | <.01 |
| Plant×Zn |  | 1 |  | 10.5 |  | <.01 |  | 0.00 |  | 0.97 |  | 0.77 |  | 0.39 |
| N×Zn |  | 2 |  | 18.6 |  | <.001 |  | 16.8 |  | <.001 |  | 0.62 |  | 0.54 |
| Plant×N×Zn |  | 2 |  | 14.7 |  | <.001 |  | 15.7 |  | <.001 |  | 4.10 |  | 0.03 |
| Source of Variation (Treatment) |  | DF |  | pH at grain filling stage | | |  | pH at mature stage | | |  |  | | |
|  |  |  |  | F |  | P |  | F |  | P |  |  |  |  |
| Plant |  | 1 |  | 241 |  | <.001 |  | 147 |  | <.001 |  |  |  |  |
| N application |  | 2 |  | 6.87 |  | <.01 |  | 19.8 |  | <.001 |  |  |  |  |
| Zn application |  | 1 |  | 29.4 |  | <.001 |  | 4.46 |  | 0.05 |  |  |  |  |
| Plant×N |  | 2 |  | 19.7 |  | <.001 |  | 6.30 |  | 0.01 |  |  |  |  |
| Plant×Zn |  | 1 |  | 34.2 |  | <.001 |  | 14.5 |  | <.001 |  |  |  |  |
| N×Zn |  | 2 |  | 25.7 |  | <.001 |  | 23.8 |  | <.001 |  |  |  |  |
| Plant×N×Zn |  | 2 |  | 3.34 |  | 0.05 |  | 1.88 |  | 0.17 |  |  |  |  |

**Table S3** Three-way analysis of variance (ANOVA) of the effects of plants, Zn and N application, as well as their interactions on the chemical fractions of Zn concentration in soil of winter wheat (*Triticum aestivum* cv Yunong 202), with three N application rates (0.05, 0.2 and 0.4 g N kg^-1^ soil) and two Zn application rates (0 and 10 mg Zn kg^-1^ soil), without and with plants at different growth stages.

| Source of Variation (Treatment) |  | DF |  | Exchangeable Zn concentration at tillering stage | | |  | Exchangeable Zn concentration at jointing stage | | |  | Exchangeable Zn concentration at grain filling stage | | |
| --- | --- | --- | --- | --- | --- | --- | --- | --- | --- | --- | --- | --- | --- | --- |
|  |  |  |  | F |  | P |  | F |  | P |  | F |  | P |
| Plant |  | 1 |  | 10.4 |  | <.01 |  | 0.87 |  | 0.36 |  | 214 |  | <.001 |
| N application |  | 2 |  | 6.52 |  | 0.01 |  | 13.7 |  | <.001 |  | 57.6 |  | <.001 |
| Zn application |  | 1 |  | 2.30 |  | 0.14 |  | 0.38 |  | 0.55 |  | 8.90 |  | 0.01 |
| Plant×N |  | 2 |  | 45.9 |  | <.001 |  | 65.0 |  | <.001 |  | 103 |  | <.001 |
| Plant×Zn |  | 1 |  | 19.8 |  | <.001 |  | 1.31 |  | 0.26 |  | 1.38 |  | 0.25 |
| N×Zn |  | 2 |  | 4.91 |  | 0.02 |  | 11.6 |  | <.001 |  | 0.68 |  | 0.51 |
| Plant×N×Zn |  | 2 |  | 5.07 |  | 0.01 |  | 2.92 |  | 0.07 |  | 1.17 |  | 0.33 |
| Source of Variation (Treatment) |  | DF |  | Exchangeable Zn concentration at mature stage | | |  | Loose organic-bound Zn concentration at tillering stage | | |  | Loose organic-bound Zn concentration at jointing stage | | |
|  |  |  |  | F |  | P |  | F |  | P |  | F |  | P |
| Plant |  | 1 |  | 2.83 |  | 0.11 |  | 3.10 |  | 0.09 |  | 19.4 |  | <.001 |
| N application |  | 2 |  | 1.05 |  | 0.37 |  | 240 |  | <.001 |  | 25.3 |  | <.001 |
| Zn application |  | 1 |  | 1.14 |  | 0.30 |  | 0.92 |  | 0.35 |  | 0.12 |  | 0.73 |
| Plant×N |  | 2 |  | 15.5 |  | <.001 |  | 224 |  | <.001 |  | 14.1 |  | <.001 |
| Plant×Zn |  | 1 |  | 1.47 |  | 0.24 |  | 73.9 |  | <.001 |  | 56.9 |  | <.001 |
| N×Zn |  | 2 |  | 0.67 |  | 0.52 |  | 8.77 |  | <.01 |  | 11.8 |  | <.001 |
| Plant×N×Zn |  | 2 |  | 2.32 |  | 0.12 |  | 27.3 |  | <.001 |  | 14.9 |  | <.001 |
| Source of Variation (Treatment) |  | DF |  | Loose organic-bound Zn concentration at grain filling stage | | |  | Loose organic-bound Zn concentration at mature stage | | |  | Carbonate-bound Zn concentration at tillering stage | | |
|  |  |  |  | F |  | P |  | F |  | P |  | F |  | P |
| Plant |  | 1 |  | 119 |  | <.001 |  | 108 |  | <.001 |  | 19.4 |  | <.001 |
| N application |  | 2 |  | 48.9 |  | <.001 |  | 26.7 |  | <.001 |  | 8.00 |  | <.01 |
| Zn application |  | 1 |  | 4.68 |  | 0.05 |  | 5.35 |  | 0.03 |  | 63.6 |  | <.001 |
| Plant×N |  | 2 |  | 41.8 |  | <.001 |  | 20.0 |  | <.001 |  | 13.2 |  | <.001 |
| Plant×Zn |  | 1 |  | 16.0 |  | <.001 |  | 18.1 |  | <.001 |  | 1.37 |  | 0.25 |
| N×Zn |  | 2 |  | 3.99 |  | 0.03 |  | 1.83 |  | 0.18 |  | 9.17 |  | <.01 |
| Plant×N×Zn |  | 2 |  | 6.11 |  | 0.01 |  | 7.47 |  | <.01 |  | 4.52 |  | 0.02 |
| Source of Variation (Treatment) |  | DF |  | Carbonate-bound Zn concentration at jointing stage | | |  | Carbonate-bound Zn concentration at grain filling stage | | |  | Carbonate-bound Zn concentration at mature stage | | |
|  |  |  |  | F |  | P |  | F |  | P |  | F |  | P |
| Plant |  | 1 |  | 58.8 |  | <.001 |  | 5.13 |  | 0.03 |  | 181 |  | <.001 |
| N application |  | 2 |  | 3.82 |  | 0.04 |  | 6.79 |  | <.01 |  | 0.37 |  | 0.69 |
| Zn application |  | 1 |  | 16.8 |  | <.001 |  | 12.5 |  | <.01 |  | 2.03 |  | 0.17 |
| Plant×N |  | 2 |  | 2.60 |  | 0.10 |  | 1.77 |  | 0.19 |  | 0.06 |  | 0.94 |
| Plant×Zn |  | 1 |  | 14.4 |  | <.001 |  | 1.58 |  | 0.22 |  | 10.1 |  | <.01 |
| N×Zn |  | 2 |  | 1.67 |  | 0.21 |  | 2.81 |  | 0.08 |  | 2.76 |  | 0.08 |
| Plant×N×Zn |  | 2 |  | 2.16 |  | 0.14 |  | 3.04 |  | 0.07 |  | 3.41 |  | 0.05 |
| Source of Variation (Treatment) |  | DF |  | Fe-Mn oxides-bound Zn concentration at tillering stage | | |  | Fe-Mn oxides-bound Zn concentration at jointing stage | | |  | Fe-Mn oxides-bound Zn concentration at grain filling stage | | |
|  |  |  |  | F |  | P |  | F |  | P |  | F |  | P |
| Plant |  | 1 |  | 16.0 |  | <.001 |  | 25.0 |  | <.001 |  | 7.95 |  | 0.01 |
| N application |  | 2 |  | 0.39 |  | 0.68 |  | 39.3 |  | <.001 |  | 17.2 |  | <.001 |
| Zn application |  | 1 |  | 1.39 |  | 0.25 |  | 2.22 |  | 0.15 |  | 1.80 |  | 0.19 |
| Plant×N |  | 2 |  | 2.21 |  | 0.13 |  | 80.5 |  | <.001 |  | 2.26 |  | 0.13 |
| Plant×Zn |  | 1 |  | 0.00 |  | 0.97 |  | 9.00 |  | 0.01 |  | 7.57 |  | 0.01 |
| N×Zn |  | 2 |  | 2.13 |  | 0.14 |  | 16.9 |  | <.001 |  | 13.9 |  | <.001 |
| Plant×N×Zn |  | 2 |  | 1.17 |  | 0.33 |  | 20.7 |  | <.001 |  | 16.0 |  | <.001 |
| Source of Variation (Treatment) |  | DF |  | Fe-Mn oxides-bound Zn concentration at mature stage | | |  | Tight organic-bound Zn concentration at tillering stage | | |  | Tight organic-bound Zn concentration at jointing stage | | |
|  |  |  |  | F |  | P |  | F |  | P |  | F |  | P |
| Plant |  | 1 |  | 0.42 |  | 0.52 |  | 3.59 |  | 0.07 |  | 4.85 |  | 0.04 |
| N application |  | 2 |  | 7.34 |  | <.01 |  | 0.71 |  | 0.50 |  | 6.47 |  | 0.01 |
| Zn application |  | 1 |  | 0.02 |  | 0.89 |  | 3.73 |  | 0.07 |  | 17.7 |  | <.001 |
| Plant×N |  | 2 |  | 4.60 |  | 0.02 |  | 1.67 |  | 0.21 |  | 4.46 |  | 0.02 |
| Plant×Zn |  | 1 |  | 6.24 |  | 0.02 |  | 0.53 |  | 0.47 |  | 9.90 |  | <.01 |
| N×Zn |  | 2 |  | 1.55 |  | 0.23 |  | 0.53 |  | 0.59 |  | 22.0 |  | <.001 |
| Plant×N×Zn |  | 2 |  | 1.08 |  | 0.35 |  | 1.64 |  | 0.22 |  | 6.04 |  | 0.01 |
| Source of Variation (Treatment) |  | DF |  | Tight organic-bound Zn concentration at grain filling stage | | |  | Tight organic-bound Zn concentration at mature stage | | |  | Residual Zn concentration at tillering stage | | |
|  |  |  |  | F |  | P |  | F |  | P |  | F |  | P |
| Plant |  | 1 |  | 1430 |  | <.001 |  | 24.2 |  | <.001 |  | 30.1 |  | <.001 |
| N application |  | 2 |  | 99.7 |  | <.001 |  | 7.69 |  | <.01 |  | 7.02 |  | <.01 |
| Zn application |  | 1 |  | 107 |  | <.001 |  | 0.20 |  | 0.66 |  | 11.7 |  | <.01 |
| Plant×N |  | 2 |  | 11.5 |  | <.001 |  | 0.45 |  | 0.64 |  | 28.9 |  | <.001 |
| Plant×Zn |  | 1 |  | 21.3 |  | <.001 |  | 0.59 |  | 0.45 |  | 0.04 |  | 0.84 |
| N×Zn |  | 2 |  | 31.6 |  | <.001 |  | 10.3 |  | <.001 |  | 2.71 |  | 0.09 |
| Plant×N×Zn |  | 2 |  | 58.5 |  | <.001 |  | 18.6 |  | <.001 |  | 4.96 |  | 0.02 |
| Source of Variation (Treatment) |  | DF |  | Residual Zn concentration at jointing stage | | |  | Residual Zn concentration at grain filling stage | | |  | Residual Zn concentration at mature stage | | |
|  |  |  |  | F |  | P |  | F |  | P |  | F |  | P |
| Plant |  | 1 |  | 3.02 |  | 0.09 |  | 94.2 |  | <.001 |  | 135 |  | <.001 |
| N application |  | 2 |  | 6.24 |  | 0.01 |  | 14.4 |  | <.001 |  | 4.15 |  | 0.02 |
| Zn application |  | 1 |  | 35.9 |  | <.001 |  | 6.18 |  | 0.02 |  | 9.44 |  | 0.01 |
| Plant×N |  | 2 |  | 2.31 |  | 0.12 |  | 0.65 |  | 0.53 |  | 2.77 |  | 0.08 |
| Plant×Zn |  | 1 |  | 1.22 |  | 0.28 |  | 0.06 |  | 0.81 |  | 4.13 |  | 0.05 |
| N×Zn |  | 2 |  | 1.34 |  | 0.28 |  | 2.98 |  | 0.07 |  | 0.78 |  | 0.47 |
| Plant×N×Zn |  | 2 |  | 1.11 |  | 0.35 |  | 1.62 |  | 0.22 |  | 3.16 |  | 0.06 |

**Table S4** Three-way analysis of variance (ANOVA) of the effects of plants, Zn and N application, as well as their interactions on the proportion of chemical fractions of Zn in soil of winter wheat (*Triticum aestivum* cv Yunong 202), with three N application rates (0.05, 0.2 and 0.4 g N kg^-1^ soil) and two Zn application rates (0 and 10 mg Zn kg^-1^ soil), without and with plants at different growth stages.

| Source of Variation (Treatment) |  | DF |  | Exchangeable Zn proportion at tillering stage | | |  | Exchangeable Zn proportion at jointing stage | | |  | Exchangeable Zn proportion at grain filling stage | | |
| --- | --- | --- | --- | --- | --- | --- | --- | --- | --- | --- | --- | --- | --- | --- |
|  |  |  |  | F |  | P |  | F |  | P |  | F |  | P |
| Plant |  | 1 |  | 41.4 |  | <.01 |  | 0.06 |  | 0.81 |  | 218 |  | <.001 |
| N application |  | 2 |  | 0.86 |  | 0.44 |  | 24.4 |  | <.001 |  | 29.9 |  | <.001 |
| Zn application |  | 1 |  | 1.01 |  | 0.32 |  | 11.6 |  | <.01 |  | 0.01 |  | 0.93 |
| Plant×N |  | 2 |  | 5.34 |  | 0.01 |  | 58.6 |  | <.001 |  | 23.5 |  | <.001 |
| Plant×Zn |  | 1 |  | 5.48 |  | 0.03 |  | 0.54 |  | 0.47 |  | 0.02 |  | 0.88 |
| N×Zn |  | 2 |  | 0.93 |  | 0.41 |  | 13.3 |  | <.001 |  | 0.08 |  | 0.92 |
| Plant×N×Zn |  | 2 |  | 6.39 |  | 0.01 |  | 7.48 |  | <.01 |  | 0.13 |  | 0.88 |
| Source of Variation (Treatment) |  | DF |  | Exchangeable Zn proportion at mature stage | | |  | Loose organic-bound Zn proportion at tillering stage | | |  | Loose organic-bound Zn proportion at jointing stage | | |
|  |  |  |  | F |  | P |  | F |  | P |  | F |  | P |
| Plant |  | 1 |  | 21.5 |  | <.001 |  | 0.63 |  | 0.43 |  | 21.0 |  | <.001 |
| N application |  | 2 |  | 1.35 |  | 0.28 |  | 61.9 |  | <.001 |  | 17.4 |  | <.001 |
| Zn application |  | 1 |  | 3.74 |  | 0.07 |  | 0.01 |  | 0.92 |  | 7.48 |  | 0.01 |
| Plant×N |  | 2 |  | 11.5 |  | <.001 |  | 83.9 |  | <.001 |  | 9.82 |  | <.001 |
| Plant×Zn |  | 1 |  | 0.01 |  | 0.91 |  | 14.1 |  | 0.001 |  | 37.7 |  | <.001 |
| N×Zn |  | 2 |  | 0.79 |  | 0.47 |  | 1.60 |  | 0.22 |  | 7.16 |  | <.01 |
| Plant×N×Zn |  | 2 |  | 1.12 |  | 0.34 |  | 3.25 |  | 0.06 |  | 6.37 |  | 0.01 |
| Source of Variation (Treatment) |  | DF |  | Loose organic-bound Zn proportion at grain filling stage | | |  | Loose organic-bound Zn proportion at mature stage | | |  | Carbonate-bound Zn proportion at tillering stage | | |
|  |  |  |  | F |  | P |  | F |  | P |  | F |  | P |
| Plant |  | 1 |  | 9.69 |  | <.01 |  | 222 |  | <.001 |  | 0.04 |  | 0.85 |
| N application |  | 2 |  | 14.5 |  | <.001 |  | 43.9 |  | <.001 |  | 5.43 |  | 0.01 |
| Zn application |  | 1 |  | 6.99 |  | 0.01 |  | 2.16 |  | 0.15 |  | 18.6 |  | <.001 |
| Plant×N |  | 2 |  | 24.3 |  | <.001 |  | 30.8 |  | <.001 |  | 28.4 |  | <.001 |
| Plant×Zn |  | 1 |  | 12.4 |  | <.01 |  | 11.8 |  | <.01 |  | 1.53 |  | 0.23 |
| N×Zn |  | 2 |  | 6.29 |  | 0.01 |  | 2.14 |  | 0.14 |  | 1.44 |  | 0.26 |
| Plant×N×Zn |  | 2 |  | 8.58 |  | <.01 |  | 7.75 |  | <.01 |  | 1.11 |  | 0.35 |
| Source of Variation (Treatment) |  | DF |  | Carbonate-bound Zn proportion at jointing stage | | |  | Carbonate-bound Zn proportion at grain filling stage | | |  | Carbonate-bound Zn proportion at mature stage | | |
|  |  |  |  | F |  | P |  | F |  | P |  | F |  | P |
| Plant |  | 1 |  | 59.0 |  | <.001 |  | 22.4 |  | <.001 |  | 96.4 |  | <.001 |
| N application |  | 2 |  | 2.95 |  | 0.07 |  | 0.44 |  | 0.65 |  | 0.26 |  | 0.78 |
| Zn application |  | 1 |  | 0.00 |  | 0.95 |  | 3.35 |  | 0.08 |  | 0.42 |  | 0.52 |
| Plant×N |  | 2 |  | 5.78 |  | 0.01 |  | 1.51 |  | 0.24 |  | 0.54 |  | 0.59 |
| Plant×Zn |  | 1 |  | 12.6 |  | <.01 |  | 1.60 |  | 0.22 |  | 7.80 |  | 0.01 |
| N×Zn |  | 2 |  | 3.85 |  | 0.04 |  | 2.25 |  | 0.13 |  | 2.17 |  | 0.14 |
| Plant×N×Zn |  | 2 |  | 5.46 |  | 0.01 |  | 4.52 |  | 0.02 |  | 2.68 |  | 0.09 |
| Source of Variation (Treatment) |  | DF |  | Fe-Mn oxides-bound Zn c proportion at tillering stage | | |  | Fe-Mn oxides-bound Zn proportion at jointing stage | | |  | Fe-Mn oxides-bound Zn proportion at grain filling stage | | |
|  |  |  |  | F |  | P |  | F |  | P |  | F |  | P |
| Plant |  | 1 |  | 2.08 |  | 0.16 |  | 12.1 |  | <.01 |  | 8.33 |  | <.01 |
| N application |  | 2 |  | 1.91 |  | 0.17 |  | 31.4 |  | <.001 |  | 26.0 |  | <.001 |
| Zn application |  | 1 |  | 0.63 |  | 0.43 |  | 22.3 |  | <.001 |  | 2.25 |  | 0.15 |
| Plant×N |  | 2 |  | 0.15 |  | 0.86 |  | 32.9 |  | <.001 |  | 5.62 |  | 0.01 |
| Plant×Zn |  | 1 |  | 0.00 |  | 0.95 |  | 10.2 |  | <.01 |  | 1.86 |  | 0.19 |
| N×Zn |  | 2 |  | 0.49 |  | 0.62 |  | 4.91 |  | 0.02 |  | 8.08 |  | <.01 |
| Plant×N×Zn |  | 2 |  | 1.55 |  | 0.23 |  | 13.4 |  | <.001 |  | 9.43 |  | 0.001 |
| Source of Variation (Treatment) |  | DF |  | Fe-Mn oxides-bound Zn proportion at mature stage | | |  | Tight organic-bound Zn proportion at tillering stage | | |  | Tight organic-bound Zn proportion at jointing stage | | |
|  |  |  |  | F |  | P |  | F |  | P |  | F |  | P |
| Plant |  | 1 |  | 27.7 |  | <.001 |  | 2.40 |  | 0.13 |  | 1.74 |  | 0.20 |
| N application |  | 2 |  | 2.59 |  | 0.10 |  | 3.29 |  | 0.05 |  | 8.15 |  | <.01 |
| Zn application |  | 1 |  | 4.76 |  | 0.04 |  | 1.20 |  | 0.28 |  | 55.0 |  | <.001 |
| Plant×N |  | 2 |  | 2.77 |  | 0.08 |  | 9.34 |  | <.01 |  | 2.84 |  | 0.08 |
| Plant×Zn |  | 1 |  | 16.8 |  | <.001 |  | 0.33 |  | 0.57 |  | 4.84 |  | 0.04 |
| N×Zn |  | 2 |  | 1.21 |  | 0.32 |  | 3.46 |  | 0.05 |  | 18.8 |  | <.001 |
| Plant×N×Zn |  | 2 |  | 2.25 |  | 0.13 |  | 1.50 |  | 0.24 |  | 4.49 |  | 0.02 |
| Source of Variation (Treatment) |  | DF |  | Tight organic-bound Zn proportion at grain filling stage | | |  | Tight organic-bound Zn proportion at mature stage | | |  | Residual Zn proportion at tillering stage | | |
|  |  |  |  | F |  | P |  | F |  | P |  | F |  | P |
| Plant |  | 1 |  | 229 |  | <.001 |  | 7.30 |  | 0.01 |  | 1.02 |  | 0.32 |
| N application |  | 2 |  | 12.3 |  | <.001 |  | 10.3 |  | <.001 |  | 29.6 |  | <.001 |
| Zn application |  | 1 |  | 16.2 |  | <.001 |  | 8.55 |  | <.01 |  | 2.76 |  | 0.11 |
| Plant×N |  | 2 |  | 7.11 |  | <.01 |  | 1.66 |  | 0.21 |  | 62.3 |  | <.001 |
| Plant×Zn |  | 1 |  | 5.08 |  | 0.03 |  | 6.49 |  | 0.02 |  | 10.3 |  | <.01 |
| N×Zn |  | 2 |  | 11.9 |  | <.001 |  | 13.3 |  | <.001 |  | 1.93 |  | 0.17 |
| Plant×N×Zn |  | 2 |  | 18.7 |  | <.001 |  | 21.7 |  | <.001 |  | 0.28 |  | 0.76 |
| Source of Variation (Treatment) |  | DF |  | Residual Zn proportion at jointing stage | | |  | Residual Zn proportion at grain filling stage | | |  | Residual Zn proportion at mature stage | | |
|  |  |  |  | F |  | P |  | F |  | P |  | F |  | P |
| Plant |  | 1 |  | 36.0 |  | <.001 |  | 1.73 |  | 0.20 |  | 22.8 |  | <.001 |
| N application |  | 2 |  | 5.70 |  | <.01 |  | 9.01 |  | <.01 |  | 20.6 |  | <.001 |
| Zn application |  | 1 |  | 11.8 |  | <.01 |  | 1.73 |  | 0.20 |  | 0.00 |  | 0.97 |
| Plant×N |  | 2 |  | 8.55 |  | <.01 |  | 10.0 |  | <.001 |  | 9.96 |  | <.001 |
| Plant×Zn |  | 1 |  | 5.43 |  | 0.03 |  | 9.09 |  | <.01 |  | 7.90 |  | <.01 |
| N×Zn |  | 2 |  | 7.28 |  | <.01 |  | 7.37 |  | <.01 |  | 1.16 |  | 0.33 |
| Plant×N×Zn |  | 2 |  | 0.06 |  | 0.95 |  | 8.75 |  | <.01 |  | 0.50 |  | 0.61 |
